# Comparative Moss-Bag Biomonitoring of Multielement Signatures in Anthropogenic Underground Systems

**DOI:** 10.3390/molecules31203727

**Published:** 2026-10-17

**Authors:** Paweł Świsłowski, Grzegorz Kłys, Aleksandra Wilk, Joanna Chwiej, Małgorzata Rajfur

**Affiliations:** 1 Institute of Biology, University of Opole, Kominka 6, 6a, 45-032 Opole, Poland; gklys@uni.opole.pl (G.K.); malgorzata.rajfur@uni.opole.pl (M.R.); 2 Faculty of Physics and Applied Computer Science, AGH University of Krakow, Al. Mickiewicza 30, 30-059 Krakow, Poland; alewilk@agh.edu.pl (A.W.); joanna.chwiej@fis.agh.edu.pl (J.C.)

**Keywords:** active moss biomonitoring, trace elements, multielement signature, deposition-related signal, anthropogenic underground system

## Abstract

Anthropogenic underground systems are challenging to monitor because restricted ventilation, particle retention, resuspension and spatial heterogeneity generate complex elemental patterns. We tested whether controlled active moss-bag biomonitoring supports comparative profiling of multielement signatures in the Historic Silver Mine in Tarnowskie Góry (TG) and the Boryszyn Loop of the Międzyrzecz Fortified Region (MRU). *Pleurozium schreberi* and *Dicranum polysetum* were co-deployed at 20 points per site for 90 days; formal between-system inference was restricted to 64 moss-bag samples from 32 underground exposure points. Profiles included Mn, Fe, Cu, Zn, Pb and Hg; Ni and Cd were excluded from continuous inference because of censoring. We analysed net concentration changes relative to corresponding initial values using PCA, PERMANOVA/PERMDISP, mixed-effects models, and Procrustes analysis, with sensitivity analyses based on final concentrations and dominant-element exclusion. Multielement signatures differed between MRU and TG for both biomonitors (PERMANOVA R^2^ = 0.367 and 0.318; both *p* < 0.001), without significant dispersion differences. MRU showed a stronger Mn signal, whereas TG showed stronger Cu, Zn and Hg signals. Site discrimination persisted after simultaneously excluding Mn and Hg, and cross-species Procrustes correlations were 0.783 and 0.771. These results support moss-bag biomonitoring for comparative multielement profiling, while inference remains limited to deposition-related signals rather than direct source attribution.

## 1. Introduction

Anthropogenic underground systems, including historical mines, adits, tunnels and military fortifications, represent challenging environments for monitoring spatial variation in airborne elemental contaminants. Aerosol transport, deposition and resuspension in subterranean spaces are strongly influenced by corridor geometry, restricted or variable ventilation, microclimatic conditions and human activity, producing spatial patterns that may differ substantially from those observed in open-air environments [1,2,3,4]. Recent investigations of cave and grotto environments have shown that local microclimate and human presence can modify particulate-matter concentrations, transport, and composition, emphasising the need for spatially resolved approaches that can characterise heterogeneous underground conditions [2,3,4]. However, comparative analysis of multielement patterns across contrasting anthropogenic underground systems remains poorly explored.

Moss-bag biomonitoring offers a complementary strategy for obtaining time-integrated elemental information where dense instrumental networks are difficult to deploy. The technique provides a defined exposure period and allows deployment according to a controlled experimental design, although methodological comparability depends strongly on moss preparation, bag construction, and exposure conditions [5]. Moss-bag elemental responses integrate several processes, including particle interception, sorption, retention and potential losses from the initial material, and should therefore not be interpreted as direct measurements of atmospheric deposition flux [6,7]. Applications in indoor and other anthropogenically influenced environments have shown that moss bags can record spatial and temporal variation in elemental exposure [8,9]. Moreover, direct comparison with PM10 filter monitoring has shown that moss bags and conventional collectors provide different but complementary representations of the atmospheric elemental signal, reflecting differences in sampling matrix and temporal integration [10].

Environmental interpretation can be extended beyond element-by-element comparisons by considering the joint structure of multielement data. Multivariate analysis of elemental profiles has been used in moss biomonitoring to recognise structured differences among environmental settings and to identify groups of co-varying elements [11]. In the present study, we use the term multielement signature operationally to describe the joint relative pattern of elemental responses recorded by exposed moss bags. It does not imply a unique source fingerprint or direct source attribution. This distinction is particularly important in underground systems, where particle transport, surface interactions and resuspension may modify the signal recorded by a biomonitor independently of the original emission source [1,3].

The biological matrix itself may additionally modulate the recorded elemental pattern. Moss morphology, surface properties and retention characteristics can influence pollutant interception and elemental accumulation, and species-specific responses have been reported in active moss biomonitoring studies [7,9,12]. Consequently, using two morphologically distinct species provides a way to test whether an environmental multielement signature is robust to biomonitor selection. *Pleurozium schreberi* and *Dicranum polysetum* were therefore treated here as two biological biomonitoring matrices exposed simultaneously to the same local conditions. The central question was not whether both species accumulated identical concentrations, but whether they preserved the same between-system contrast and comparable multivariate structure.

The present study compares two anthropogenic underground systems of contrasting origin: the historical mining system of the Historic Silver Mine in Tarnowskie Góry (TG) and the military fortification system of the Boryszyn Loop in the Międzyrzecz Fortified Region (MRU). Their different histories, construction characteristics and environmental settings provide a suitable empirical contrast for evaluating whether controlled active moss biomonitoring can discriminate multielement signatures between underground systems without assuming that either site universally represents its broader system category. This objective differs from our previous underground study, which combined soil, naturally occurring moss and moss bags within a single bat hibernation site to evaluate local spatial patterns and elemental hotspots [13]. The present study uses independent sites, samples, and data; includes only actively exposed moss biomonitors; and shifts the analytical focus from within-site contamination assessment to comparative multielement profiling between two anthropogenic underground systems.

Accordingly, this study investigated whether controlled moss-bag exposure can provide consistent multielement signatures that discriminate two contrasting anthropogenic underground systems and whether these signatures remain consistent across two moss biomonitors. Three hypotheses were tested: (H1) TG and MRU differ in their multielement signatures recorded by actively exposed mosses; (H2) the between-system contrast is expressed by a structured combination of elemental signals rather than being attributable to a single dominant element; and (H3) biomonitor species may modify the magnitude of individual elemental responses without fundamentally altering the between-system multielement pattern. We also evaluated the robustness of the resulting discrimination using alternative response definitions, dominant-element exclusion, and cross-species concordance analyses. The broader objective was to assess active moss-bag biomonitoring as a comparative framework for multielement characterisation of anthropogenic underground systems rather than as a tool for direct source identification or universal classification of mines and fortifications.

## 2. Results

### 2.1. Analytical Performance and Dataset Definition

We recovered all 80 deployed moss bags, providing 640 sample–element records for the eight analysed elements. Analytical accuracy assessed using BCR-482 lichen certified reference material showed relative differences between measured and certified concentrations ranging from −13.0% for Ni to −3.9% for Mn (Table S2). The primary inferential dataset comprised 64 underground moss-bag samples, corresponding to 384 quantitative sample–element observations for Mn, Fe, Cu, Zn, Pb and Hg. Table 1 summarises final concentrations (Cexp) and corresponding net concentration changes (NE), with complete descriptive statistics in Table S4.

Initial elemental concentrations varied among the four Site × Species starting materials, and NE was therefore calculated relative to the corresponding C_0_ value. Ni and Cd were not included in continuous multivariate or mixed-model inference because of analytical censoring. Ni occurred at or below the operational LOQ in 80.6% of MRU and 82.1% of TG underground observations (Fisher’s exact test, *p* = 1.00). Cd censoring was strongly asymmetric, occurring in 86.1% of MRU but only 7.14% of TG observations (*p* < 0.001). We retained these observations for descriptive analysis but did not treat them as precise continuous concentrations. Initial values and censoring frequencies are summarised in Table S3.

### 2.2. Chemometric Structure of Multielement Signatures

**Figure 1:**
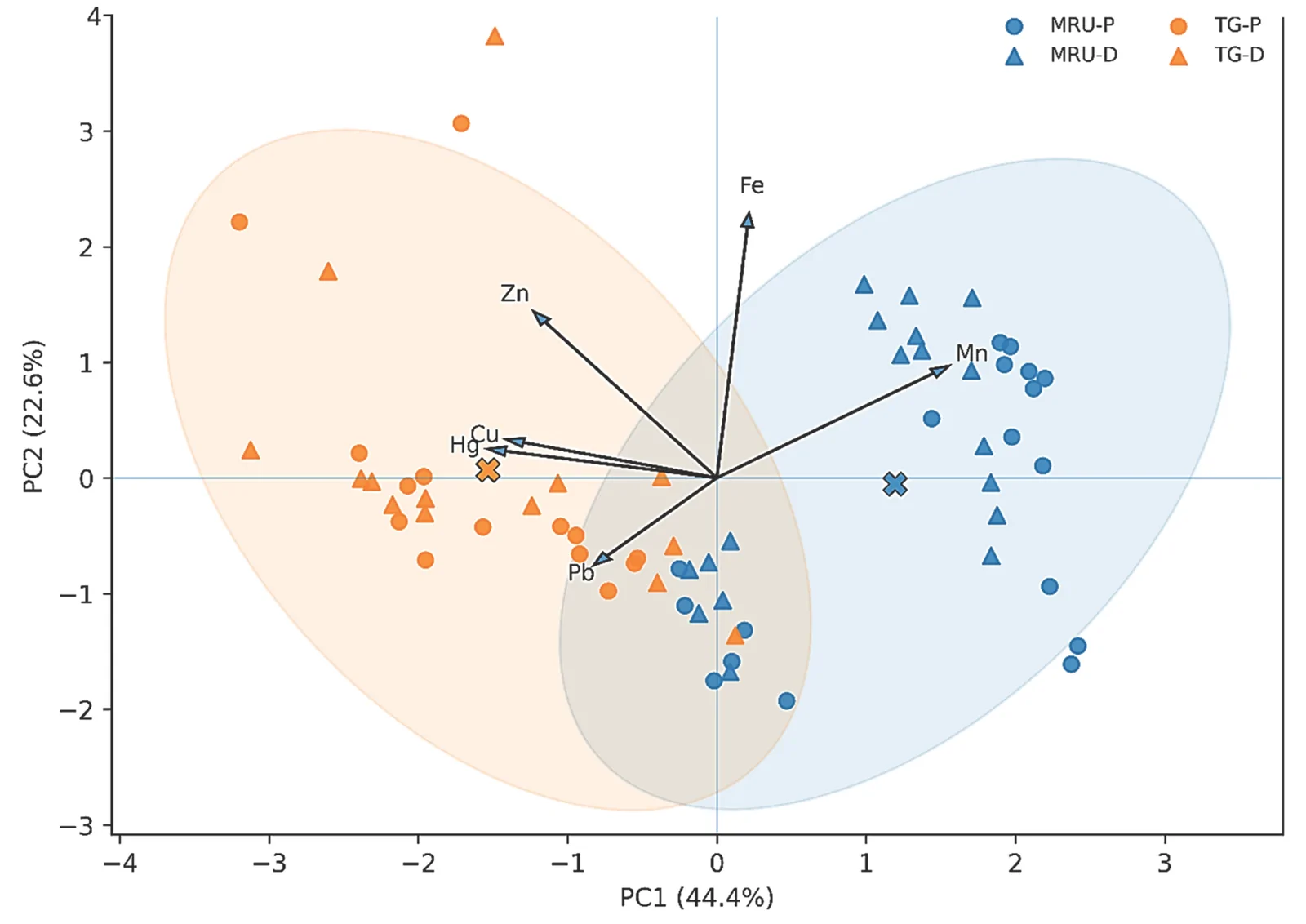
PCA ordination of centred and standardised six-element NE profiles for underground MRU and TG samples. Colours denote the underground system, symbols denote biomonitor species, crosses indicate system centroids, ellipses are descriptive, and vectors represent element loadings. PC1 and PC2 explain 44.4% and 22.6% of total variance, respectively.

**Figure 2:**
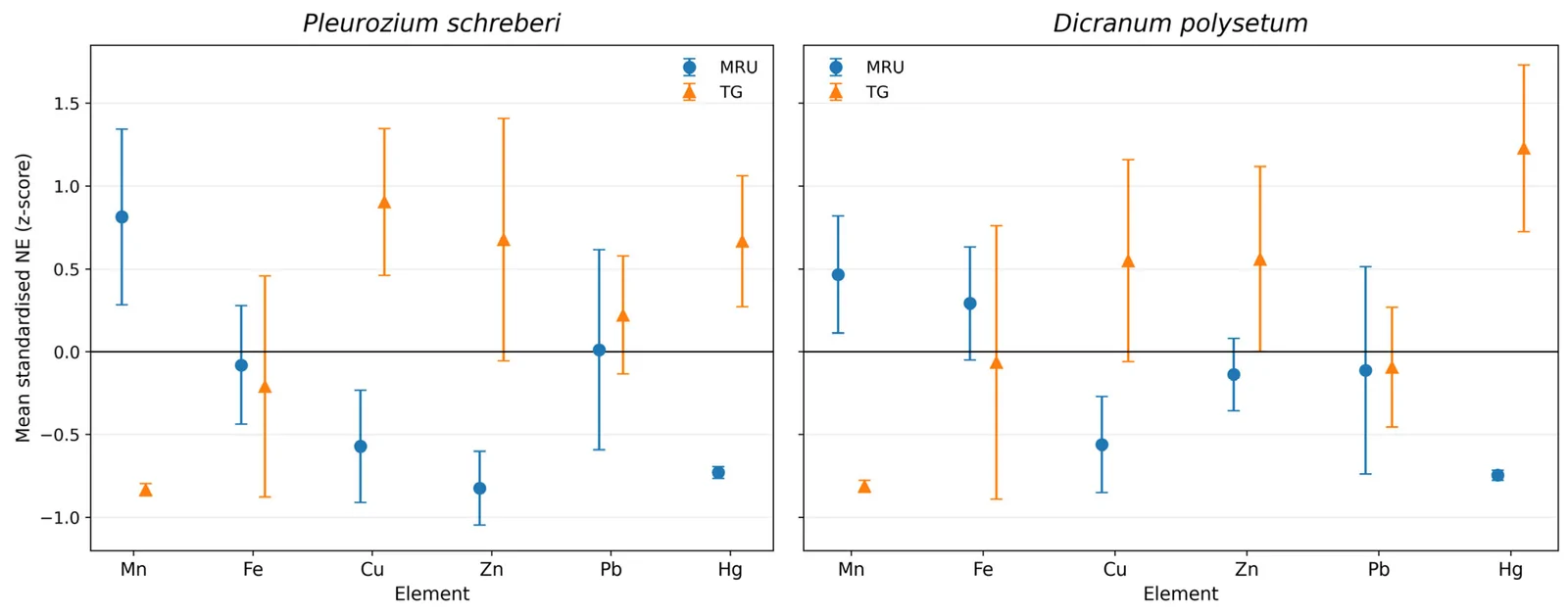
Standardised element-level NE profiles for the two moss biomonitors. Points show mean z-scores and bars show descriptive 95% confidence intervals for MRU and TG within each species. We standardised data jointly across all underground samples, separately for each element. We used PERMANOVA for formal multielement comparisons, and evaluated element-specific effects with mixed-effects models.

PCA of centred and standardised NE values showed that the main differentiation between MRU and TG occurred along PC1, which explained 44.4% of total variance; PC2 explained a further 22.6% (Figure 1). PC1 was characterised by a positive Mn loading opposed primarily by Hg and Cu, with an additional Zn contribution, whereas PC2 was dominated by Fe and, to a lesser extent, Zn (Table S5). Both moss species showed the same general between-system configuration: MRU samples were associated with a stronger Mn signal, whereas TG samples were associated with stronger Cu, Zn and Hg signals. The same directional structure was visible in the standardised group profiles (Figure 2).

PERMANOVA confirmed significant differences between the complete six-element signatures of MRU and TG for both *Pleurozium schreberi* (pseudo-F = 17.4, R^2^ = 0.367, *p* < 0.001) and *Dicranum polysetum* (pseudo-F = 14.0, R^2^ = 0.318, *p* < 0.001). PERMDISP was not significant for either species (*P. schreberi*: F = 0.0912, *p* = 0.778; *D. polysetum*: F = 0.564, *p* = 0.488), supporting the interpretation of the primary PERMANOVA results as differences in multivariate location rather than consequences of unequal within-group dispersion. Full multivariate test results are given in Table S6A.

### 2.3. Elemental Contributions to Between-System Differentiation

Element-specific mixed-effects models showed significant between-system effects for Mn, Cu, Zn and Hg after Benjamini–Hochberg correction, whereas Fe and Pb did not differ significantly (Table 2). Averaged across biomonitor species, the TG − MRU effect was −383 mg kg^−1^ for Mn (q < 0.001), indicating higher NE values in MRU, whereas effects for Cu (+1.35 mg kg^−1^), Zn (+12.2 mg kg^−1^) and Hg (+0.117 mg kg^−1^) indicated higher NE values in TG (all q < 0.001). Full fixed-effect results are provided in Table S7A.

Significant Site × Species interactions occurred for Mn (q = 0.0136) and Zn (q < 0.001). Planned simple contrasts showed that Mn NE values were higher in MRU for both biomonitors, but the between-system contrast was larger for *Pleurozium schreberi* (TG − MRU = −432 mg kg^−1^, 95% CI: −553 to −311; pHolm < 0.001) than for *Dicranum polysetum* (−335 mg kg^−1^, 95% CI: −455 to −214; pHolm < 0.001). Zn showed the opposite direction: NE values were higher in TG for both species, with a larger contrast for *P. schreberi* (+16.5 mg kg^−1^, 95% CI: 9.99–23.1; pHolm < 0.001) than for *D. polysetum* (+7.76 mg kg^−1^, 95% CI: 1.20–14.3; pHolm = 0.0219). Thus, the interactions altered the magnitude, but not the direction, of the principal Mn and Zn contrasts. The corresponding planned simple contrasts are reported in Table S7B.

### 2.4. Species-Associated Modulation and Cross-Biomonitor Consistency

Species-associated differences were element-dependent. Within MRU, *Dicranum polysetum* showed lower Mn NE than *Pleurozium schreberi* (D − P = −91.6 mg kg^−1^, 95% CI: −155 to −28.3; pHolm = 0.0121), whereas no corresponding difference was detected in TG. For Zn, *D. polysetum* showed higher NE than *P. schreberi* in MRU (+7.56 mg kg^−1^, 95% CI: 4.98–10.1; pHolm < 0.001), while the species contrast was not significant in TG. For elements without significant interactions, *D. polysetum* showed higher marginal Fe NE (+15.4 mg kg^−1^; q = 0.0252) and lower Pb NE (−0.481 mg kg^−1^; q = 0.0125); no corrected marginal species effect was detected for Cu or Hg. Element-level model and contrast details are provided in Table S7.

Paired PERMANOVA indicated a significant species-associated contribution to the complete six-element profile: species explained 8.49% of profile variation within MRU (pseudo-F = 3.16, R^2^ = 0.0849, *p* < 0.001) and 4.32% within TG (pseudo-F = 1.17, R^2^ = 0.0432, *p* = 0.0265). These estimates came from separate within-system paired analyses; we did not interpret them as direct variance partitions comparable to the between-system PERMANOVA R^2^ values. Table S6B summarises the paired multivariate results.

Despite these amplitude differences, the two biomonitors recorded a strongly concordant relative multivariate configuration. Procrustes correlations were 0.783 in MRU and 0.771 in TG, with PROTEST *p* < 0.001 in both systems. The two species therefore preserved a similar relative organisation of exposure points, although their absolute elemental responses were not quantitatively interchangeable. Cross-biomonitor concordance statistics are also reported in Table S6B.

### 2.5. Robustness of Multielement Differentiation

The between-system contrast was robust to alternative analytical definitions and to removal of dominant contributors. When profiles were expressed using final concentrations rather than NE, MRU and TG remained separated for both *Pleurozium schreberi* (R^2^ = 0.370, *p* < 0.001) and *Dicranum polysetum* (R^2^ = 0.321, *p* < 0.001), without significant PERMDISP effects.

Multielement differentiation also did not depend solely on Mn or Hg. After simultaneously excluding both elements, PERMANOVA remained significant for *P. schreberi* (pseudo-F = 9.51, R^2^ = 0.241, *p* < 0.001; PERMDISP *p* = 0.820) and *D. polysetum* (pseudo-F = 4.83, R^2^ = 0.139, *p* < 0.001; PERMDISP *p* = 0.399). All sequential single-element exclusion analyses retained significant MRU–TG PERMANOVA separation. One sensitivity variant, *D. polysetum* after exclusion of Pb, showed modest dispersion heterogeneity (PERMDISP *p* = 0.0451); this exception was not observed in the full analysis or in the central Mn + Hg exclusion analysis. Complete element-exclusion results are provided in Table S9.

Point-cluster-robust models of log-transformed final concentrations independently reproduced the direction of the main between-system effects: TG/MRU ratios were 0.279 for Mn, 1.282 for Cu, 1.410 for Zn and 3.716 for Hg (all q < 0.001), whereas Fe and Pb remained non-significant. The Zn Site × Species interaction persisted under this alternative response definition (ratio-of-ratios = 0.762, q < 0.001), whereas the Mn interaction did not, indicating that species modulation of Mn was more dependent on response definition than the corresponding between-system effect. Full sensitivity-model results are provided in Table S8B.

Point-cluster bootstrap and leave-one-point-out analyses preserved the directions of the principal Mn, Cu, Zn, and Hg between-system effects, indicating that a single exposure point did not drive the main discrimination. We retained outdoor observations only as descriptive projections onto the multivariate space defined by the underground dataset and did not use them for formal inference because their number and environmental context differed between MRU and TG. Table S8A reports point-level robustness results, and Figure S3 shows the outdoor PCA projection.

## 3. Discussion

### 3.1. Contrasting Multielement Signatures in Anthropogenic Underground Systems

The principal finding of this study was that the two anthropogenic underground systems were characterised by distinct multielement signatures rather than by a uniform increase or decrease across all analysed elements. MRU was characterised primarily by a stronger Mn signal, whereas TG showed stronger Cu, Zn and Hg signals; Fe and Pb contributed less consistently to between-system differentiation. Both *P. schreberi* and *D. polysetum* showed the same general contrast, and multivariate separation was not attributable to differences in within-group dispersion. These results support H1 and indicate that actively exposed mosses can integrate system-specific differences into a multielement response pattern rather than merely provide a set of independent concentration measurements.

Such differentiation is plausible in underground environments, where particle transport and retention are shaped by site-specific corridor geometry, air-exchange conditions, microclimate, surface interactions and human activity. Studies in caves and other subterranean environments have demonstrated that particulate-matter abundance and composition can differ substantially between underground and external environments and may respond rapidly to visitor activity and microclimatic conditions [1,3,4]. Miler et al. [3], for example, documented direct effects of tourist activity on particulate-matter concentrations in a show cave, while Yang et al. [4] demonstrated compositional differences between particles collected inside and outside a grotto. These studies provide a process-based context for the observed MRU–TG contrast, but they do not identify the sources responsible for the elemental responses recorded by the moss bags.

The particularly strong Mn signal in MRU may therefore reflect an Mn-enriched deposition within this system, but not a specific Mn source. Potential contributors include mineral particles potentially derived from the surrounding geological setting, concrete and other construction materials, corrosion products from metallic infrastructure, resuspended settled dust, and particles entering from the external environment. The central part of the MRU is characterised by a heterogeneous Quaternary geomorphological and lithostratigraphic setting [14], which supports considering a possible geogenic contribution but does not establish the origin of Mn. Studies of subterranean environments have shown that particulate matter may derive from resuspended mineral material, wall or ceiling material, infrastructure, and particles introduced from outside [3,4,15]. Airborne particles, settled dust, geological material and construction materials were not analysed in the present study; the relative contribution of these pathways cannot be resolved, and the elevated Mn signal should not be attributed to a specific source. The same caution applies to TG. The stronger Zn signal at TG is geochemically consistent with the local Zn–Pb–Ag mineralisation. The Tarnowskie Góry district belongs to the Silesian–Cracow Mississippi Valley-type ore province, where mineralisation is hosted mainly by Middle Triassic ore-bearing dolomites and includes Zn- and Pb-bearing sulphides together with secondary weathering products [16]. The Historic Silver Mine itself is developed within shallow ore-bearing dolomites, with galena as the principal ore mineral and sphalerite and wurtzite also reported locally [17]. This geological context makes a contribution of Zn-bearing mineral material to the recorded moss-bag signal plausible, although the present study cannot demonstrate its direct origin. The local geological sources considered here do not identify Cu as a major constituent of the characteristic ore assemblage; its stronger signal at TG should therefore be interpreted more cautiously as a site-associated component of the TG multielement signature rather than directly attributed to historical Zn–Pb–Ag mineralisation.

Hg requires particular caution because mosses can integrate Hg supplied through more than one atmospheric pathway. The consistently higher Hg response underground at TG is compatible with retention or secondary mobilisation within the underground system, but the present exposure design cannot distinguish particle-bound Hg from gaseous uptake. This distinction is analytically important. Meloni et al. [18] combined covered and uncovered moss bags with gaseous elemental mercury measurements in a former Hg-mining district specifically to separate aspects of Hg exposure that cannot be resolved from a single moss-bag configuration. Consequently, the present Hg result should be interpreted as a diagnostic component of the TG multielement signature rather than evidence for a defined Hg source or pathway. Cd required separate interpretation because 86.1% of MRU observations were at or below the operational LOQ, compared with only 7.14% in TG. The same LOQ applied to both systems; this asymmetry reflects a difference in the proportion of quantifiable post-exposure Cd measurements rather than in analytical sensitivity. However, initial Cd concentrations were above the LOQ in all four Site × Species materials, and moss-bag responses may include both element retention or uptake and losses from the initial material [6,7]. The Cd pattern should therefore be regarded as potentially diagnostic but not as a precise quantitative between-system contrast in the Cd-related moss-bag response.

### 3.2. Multielement Signatures Beyond Single-Element Contrasts

The study’s main analytical value lies in the joint interpretation of elemental responses. Mn and Hg were the strongest contributors to the principal multivariate gradient, but the MRU–TG distinction was not dependent on these two elements alone. After simultaneously excluding Mn and Hg, the remaining Cu–Zn–Fe–Pb profile continued to discriminate the systems for both biomonitors, with no evidence of dispersion heterogeneity in this central sensitivity analysis. Sequential exclusion of individual elements produced the same general inferential conclusion. The isolated PERMDISP result for Dicranum polysetum after excluding Pb (*p* = 0.0451) indicates that not every reduced profile behaved equally well statistically, but it does not alter the interpretation of the full profile or the predefined Mn + Hg sensitivity analysis.

This result matters because it shifts the interpretation from “MRU has more Mn and TG has more Hg” to a structured multielement contrast. The elements with the largest individual effects contribute strongly to discrimination, but they do not fully define it. Multivariate elemental approaches have been applied in moss biomonitoring to identify compositional patterns and relationships among element groups [11], and can also be extended to source apportionment when independent source information is available. In the present study, the term multielement signature is deliberately narrower: it describes a consistent relative pattern in the moss-bag response and does not imply a unique geochemical fingerprint or source identity.

The robustness analyses further strengthen this interpretation. Between-system directions for Mn, Cu, Zn, and Hg were independently reproduced when final concentrations (Cexp) were analysed without subtracting C_0_, while Fe and Pb remained non-discriminatory. Point-cluster bootstrap and leave-one-point-out analyses also preserved the principal site-level directions. The agreement between NE- and Cexp-based analyses is particularly relevant because each C_0_ estimate was derived from a single initial moss sample, with five subsamples analysed from that material. The Cexp analysis cannot remove initial differences among moss materials, but it shows that the central MRU–TG contrast was not created solely by subtracting shared initial values.

This distinction is also consistent with previous evidence that mosses and conventional particulate collectors do not represent identical analytical matrices. In a direct PM10–moss comparison, Świsłowski et al. [10] found clear multivariate separation between filter and moss profiles, indicating that mosses integrate deposition, retention and accumulation over time rather than reproduce the composition of short-term filter samples. The present study extends that concept: rather than treating the moss response as a surrogate measurement of air concentration, we use the integrated biological signal comparatively to discriminate environmental multielement patterns.

### 3.3. Biomonitor Species as a Methodological Filter of the Multielement Signal

The two moss species were not quantitatively interchangeable. Species-associated differences occurred for several individual elements, and Site × Species interactions were detected for Mn and Zn in the primary NE models. For Mn, however, the interaction did not persist when we analysed log-transformed final concentrations, whereas the Zn interaction remained significant. The evidence for species-dependent modulation is therefore stronger and more response-definition-independent for Zn than for Mn. Importantly, these differences affected response magnitude rather than reversing the main between-system directions: both species recorded a stronger Mn signal in MRU and stronger Cu, Zn and Hg signals in TG.

Species-dependent accumulation is expected because moss morphology, surface structure, water relations and particle-retention characteristics influence the quantity and form of material retained by the gametophyte [7]. Large-scale biomonitoring studies have likewise demonstrated that elemental concentrations can vary among moss species even under comparable regional exposure conditions [12,19]. More directly relevant to the present experiment, Chaligava et al. [9] compared *Pleurozium schreberi*, *Dicranum polysetum* and Sphagnum fallax under common urban–industrial exposure and found species-dependent differences for selected elements. Consequently, differences between *P. schreberi* and *D. polysetum* should be considered an intrinsic component of the biological measurement system rather than analytical noise.

At the same time, the high Procrustes concordance within both systems indicates that the two biomonitors preserved similar relative multivariate organisation of exposure points despite differences in response amplitude. This distinction is methodologically important. Quantitative interchangeability is not required for structural agreement. For comparative environmental profiling, two biological sensors may differ in accumulation efficiency while still recording the same dominant environmental contrast. The present data therefore support using multiple species to test signal stability, but not unrestricted substitution of one species for another in quantitative monitoring programmes. Standardised campaigns should retain a consistent biomonitor species or establish empirical cross-species calibration when absolute comparisons are required [12,19].

### 3.4. Analytical Value and Scope of Active-Only Moss Biomonitoring

The present study also defines what an active-only biomonitoring design can provide when passive environmental matrices are unavailable. A controlled exposure period, common bag construction, paired deployment of two biomonitors and explicit characterisation of initial material generated comparable biological sampling units across the two underground systems. Methodological standardisation is critical in moss-transplant studies because bag geometry, moss preparation, biomass loading, exposure period and sampling-network design can influence both accumulation and reproducibility [20,21,22]. Recent work on optimised moss transplants similarly emphasises that sampling design and data treatment must match the monitoring objective rather than be treated as secondary procedural details [22].

Within those constraints, active moss biomonitoring offers an integrated biological sampling strategy for environments in which extensive instrumental networks may be impractical. Indoor moss-bag experiments have already shown that active moss exposure can resolve elemental variation under confined conditions [8], while the present study extends this approach to comparative profiling of two large anthropogenic underground systems. The contribution is therefore not a new instrumental technique, but an analytical workflow linking controlled biological sampling, elemental determination and multivariate pattern analysis.

This interpretation is particularly relevant to the selected environmental analytical chemistry context. The information produced by moss bags is not equivalent to air-volume-normalised aerosol concentrations, deposition fluxes or direct measurements of settled dust. Previous comparison with PM10 filters confirms that these matrices capture different aspects of the atmospheric elemental signal [10]. Active moss bags should therefore be regarded as complementary time-integrating biological collectors rather than substitutes for conventional atmospheric samplers.

The approach may be useful in historical mines, adits, fortifications, tunnels and underground heritage sites, particularly where spatial replication is desirable, and installation of permanent instrumentation is constrained. This potential transferability remains a proposition to be tested, not a result demonstrated by the present two-site design. Future studies should replicate the workflow across multiple systems of each type and combine moss-bag exposure with direct aerosol, settled-dust and material analyses. Such integration would link the comparative signature identified by biomonitoring more directly to environmental processes and potential sources.

The biological context is also relevant. Both investigated systems serve as underground refugia for bats, yet the moss-bag protocol required no capture, handling, or biological sampling of vertebrates. This does not demonstrate the absence of ecological disturbance, but it shows that the analytical sampling strategy can be implemented without directly manipulating the animals occupying these systems. That feature may be useful when designing monitoring in environmentally or conservation-sensitive underground sites.

### 3.5. Limitations and Interpretation Boundaries

Several limitations determine the level of inference supported. First, each underground-system category was represented by a single site. MRU and TG therefore constitute two contrasting case systems, and the Site effect cannot be interpreted as a general difference between military fortifications and historical mines. The exposure campaigns both lasted 90 days but did not fully overlap in calendar time, meaning site identity, season, ventilation conditions, and other temporal factors cannot be fully separated. Visitor activity also differed temporally between the two campaigns and therefore cannot be separated from the overall Site effect. The uneven number and environmental setting of outdoor points further preclude balanced underground–surface inference; those samples are appropriately treated only as descriptive context.

Second, the study quantifies elemental responses in actively exposed mosses rather than total environmental contamination or atmospheric deposition flux. Only one initial moss sample was available for each Site × Species combination, although each C_0_ value was based on five subsamples from that material. The resulting SD therefore characterises within-sample variability. The independent analysis of Cexp reproduced the principal between-system directions and therefore reduces concern that the main findings were an artefact of C_0_ subtraction, but it does not eliminate initial differences in moss composition. Future experiments should include replicated independent initial moss samples within every Site × Species preparation batch.

Third, extensive censoring prevented inclusion of Ni and Cd in continuous multielement profiling. In particular, the strongly asymmetric Cd censoring between MRU and TG may itself contain environmental information, but the present analytical resolution does not support treating LOQ-coded values as precise continuous concentrations. A method with lower quantification limits could determine whether Cd provides additional discriminatory information.

Finally, no simultaneous analyses of airborne particulate matter, settled dust, construction materials, particle mineralogy or gaseous Hg were available. The study therefore cannot identify elemental sources, distinguish particulate from gaseous Hg pathways, quantify deposition fluxes, assess long-term accumulation in the underground environment or infer ecological or human-health risk. These are not deficiencies that invalidate the comparative biomonitoring result; they define its proper scope. The supported conclusion is narrower: under the applied exposure design, two moss biomonitors recorded consistently different multielement signatures in MRU and TG, and the distinction remained robust to alternative analytical definitions and removal of the dominant contributors.

## 4. Materials and Methods

### 4.1. Study Systems and Exposure Design

The study was conducted in two anthropogenic underground systems of contrasting origin: the Boryszyn Loop in the Międzyrzecz Fortified Region (MRU; 52.355307° N, 15.466700° E), representing a military fortification system, and the Historic Silver Mine in Tarnowskie Góry (TG; 50.426111° N, 18.849894° E), representing a historical mining system. Both systems contain tourist routes and also serve as bat hibernation sites; however, no bats or other vertebrates were captured, handled, tagged, sampled, or deliberately disturbed during the study [23,24]. Both systems are accessible to tourists, but visitor regimes differed during the exposure periods. TG operates a tourist route throughout the year, whereas regular underground walking tours at MRU are conducted seasonally from April to October. Figure S1 provides a schematic overview of the study and analytical workflow, and Figure S2 shows detailed exposure networks with site photographs.

We established twenty exposure points at each site. MRU comprised 18 underground points and two outdoor points located in woodland near the entrance, whereas TG comprised 14 underground points and six outdoor points located around the above-ground infrastructure. *Pleurozium schreberi* and *Dicranum polysetum* were co-deployed at each point, providing paired observations within each physical exposure location. Both exposure campaigns lasted 90 days, from February to May 2025 at MRU and from March to June 2025 at TG. The number and environmental setting of outdoor points differed between sites; outdoor samples were used only as descriptive context. Formal between-system inference was restricted to the 32 underground exposure points, corresponding to 64 moss-bag samples.

### 4.2. Moss Material, Preparation and Deployment

*Pleurozium schreberi* (P) and *Dicranum polysetum* (D) were collected from forest locations away from direct road and built-up-area influence, following the general sampling criteria of the ICP Vegetation protocol [25]. We mechanically cleaned the collected material to remove foreign material, conditioned it for 60 min in demineralised water (conductivity ≤ 1.0 µS cm^−1^), and dried it at 22–24 °C for 20–24 h, following the preparation procedure described previously [26].

Flat nylon bags (10 × 10 cm; 2 mm mesh) were filled with 2.00 g dry moss, corresponding to a biomass-to-external-area ratio of 10.0 mg cm^−2^. Bag dimensions, mesh size and biomass loading were kept constant throughout the experiment to minimise exposure-design variability [20,21]. At each exposure point, bags containing the two moss species were positioned side by side at approximately 1.5–2.0 m above the floor and at least 10 cm from walls or other surfaces.

For each Site × Species combination, one prepared, unexposed initial moss sample was available. Five subsamples were analysed from each initial sample, and the corresponding C_0_ value used to calculate NE was the mean of these determinations. Mean C_0_ values and the corresponding sample SDs, calculated across the five subsample determinations, are provided in Table S3A.

### 4.3. Sample Preparation and Elemental Determination

After exposure, we selected green gametophyte material for elemental analysis [27]. Green gametophyte remained available after the 90-day exposure; however, moss viability was not assessed physiologically [28]. Post-exposure changes in total moss biomass or gametophyte morphology were not quantified. For the determination of Mn, Fe, Ni, Cu, Zn, Cd and Pb, an 800 mg subsample was transferred to a digestion vessel containing 10 mL of 65% HNO_3_ and 6 mL of 30% H_2_O_2_ and left for 24 h before microwave digestion using a Speedwave XPERT system (Berghof, Eningen, Germany). The digestion programme consisted of heating to 145 °C over 10 min, followed by 5 min at 145 °C; heating to 190 °C over 5 min, followed by 10 min at 190 °C; and cooling to 50 °C over 20 min. Microwave power was set to 90% during the heating stages, with a pressure limit of 40 bar. The digests were quantitatively transferred to 20 mL volumetric flasks and diluted with deionised water.

We determined Mn, Fe, Ni, Cu, Zn, Cd, and Pb in the mineralised samples by flame atomic absorption spectrometry (F-AAS) using an iCE 3500 spectrometer (Thermo Fisher Scientific, Waltham, MA, USA). Mercury was determined independently and directly in dry, non-mineralised moss material using an AMA 254 mercury analyser (Altec, Prague, Czech Republic). Element concentrations were expressed as mg kg^−1^ dry matter. We analysed each sample in three instrumental replicates. We calibrated the F-AAS system using analytical standard solutions supplied by ANALYTIKA Ltd. (Prague, Czech Republic). Table S1 summarises analytical parameters for the F-AAS and direct AMA-254 pathways.

### 4.4. Analytical Quality Assurance and Data Handling

We evaluated analytical performance using certified reference material BCR-482 lichen (n = 5). Measured concentrations differed from certified values by −13.0% to −3.9% across the analysed elements (Table S2). Instrumental limits of detection and quantification were determined separately for the F-AAS analytical-solution pathway and for the direct-solid AMA-254 Hg determination and are reported in Table S1. For F-AAS, the interpretation of solution-based limits additionally accounts for the 0.800 g sample mass and final digest volume of 20 mL.

Ni and Cd required specific treatment because a substantial proportion of underground observations occurred at or below the operational quantification limit. Ni censoring was similar between systems (80.6% in MRU and 82.1% in TG; Fisher’s exact test, *p* = 1.00), whereas Cd censoring was strongly asymmetric (86.1% in MRU and 7.14% in TG; Fisher’s exact test, *p* < 0.001). LOQ-coded observations were therefore not treated as precise continuous concentrations. Ni and Cd were retained for descriptive censoring summaries but excluded from mixed-effects models and continuous multivariate analyses. The quantitative multielement signature was consequently based on Mn, Fe, Cu, Zn, Pb and Hg. Censoring frequencies are reported in Table S3B.

To account for differences in the initial elemental composition of the moss material, we calculated net concentration change as NE = Cexp − C_0_, where Cexp is the final concentration after exposure, and C_0_ is the corresponding Site × Species initial value. Negative NE values were retained and indicate only that Cexp was lower than the corresponding C_0_; they were not interpreted as negative deposition. Both campaigns lasted exactly 90 days; division of NE by exposure duration would apply the same constant scaling to all observations and was therefore not used in inferential analyses. Final concentrations (Cexp) were retained as an alternative response definition for sensitivity analysis independent of C_0_ subtraction.

### 4.5. Statistical and Chemometric Analysis

The primary inferential dataset comprised the 64 moss bags exposed at underground points. Descriptive statistics were calculated for final concentrations (Cexp) and net concentration changes (NE) for Mn, Fe, Cu, Zn, Pb and Hg.

For each element, a linear mixed-effects model was fitted as NE ~ Site × Species + (1|Point_ID), with Site and Species as fixed effects and physical exposure point as a random intercept. Point_ID was unique across the complete dataset and accounted for the paired deployment of *Pleurozium schreberi* and *Dicranum polysetum* at the same location [29]. We compared equal and site-specific residual-variance structures using Akaike’s information criterion (AIC), and retained the more complex structure when AIC decreased by at least two units [30]. False discovery rate was controlled separately across the six Site, six Species and six Site × Species tests using the Benjamini–Hochberg procedure [31]. We used point-level cluster bootstrap and leave-one-point-out analyses to assess robustness to non-normal residual behaviour and influential exposure locations.

If the Site × Species interaction remained significant after Benjamini–Hochberg correction, planned TG–MRU contrasts were estimated separately for each species and P–D contrasts separately for each system; *p*-values within each pair of contrasts were adjusted using the Holm method [32]. Marginal species effects were interpreted only when the Site × Species interaction was not significant.

We conducted principal component analysis (PCA) on centred and standardised NE values for Mn, Fe, Cu, Zn, Pb, and Hg to examine the structure of the multielement signatures [33]. We tested differences between the complete MRU and TG signatures separately for each moss species using PERMANOVA based on Euclidean distances among standardised profiles and 9999 permutations [34]. PERMDISP was used to determine whether significant PERMANOVA results reflected differences in multivariate location rather than unequal within-group dispersion [35].

We evaluated the effect of biomonitor species on the complete multielement profile separately within MRU and TG using paired PERMANOVA. Permutations were restricted to the two species observations belonging to the same Point_ID, preserving the paired study design; a full enumeration of possible within-pair label swaps was used [36].

We evaluated cross-species structural concordance separately within MRU and TG using Procrustes analysis with PROTEST, comparing the relative configuration of paired exposure points between *Pleurozium schreberi* and *Dicranum polysetum* [37]. To evaluate whether between-system discrimination depended disproportionately on individual elements, we repeated PERMANOVA/PERMDISP analyses after sequentially excluding each element and after simultaneously excluding Mn and Hg, the strongest contributors to PC1.

We also assessed robustness to response definition using log-transformed final concentrations, log(Cexp), avoiding subtraction of the shared C_0_ value. For each element, Site × Species models were fitted with covariance clustered by physical exposure point, and marginal TG/MRU ratios and Site × Species ratio-of-ratios were estimated with cluster-robust standard errors [38]. We projected outdoor samples onto the PCA space defined by underground observations using the centring, scaling, and loading parameters fitted to the underground dataset, and interpreted the results descriptively only.

All statistical analyses and graphical outputs were generated in R version 4.6.1 (R Foundation for Statistical Computing, Vienna, Austria), using packages including readxl, dplyr, tidyr, nlme, emmeans, vegan, permute and ggplot2. Statistica 13.3 (TIBCO Software Palo Alto, CA, USA) was used as a supplementary tool to verify descriptive statistics and PCA outputs.

## 5. Conclusions

Controlled moss-bag exposure differentiated the multielement signatures of two contrasting anthropogenic underground systems. MRU was characterised primarily by a stronger Mn signal, whereas TG showed stronger Cu, Zn and Hg signals. Both Pleurozium schreberi and Dicranum polysetum reproduced the distinction, and it persisted after removing the dominant Mn and Hg contributions, showing that the between-system contrast reflected a broader multielement structure rather than a single-element effect.

Biomonitor species influenced the magnitude of selected elemental responses, but did not reverse the principal MRU–TG pattern. Cross-species concordance therefore indicates that the environmental signal was structurally stable across two morphologically distinct moss biomonitors, while the observed quantitative differences confirm that the species should not be treated as fully interchangeable.

The main contribution of this study is methodological: active moss biomonitoring can be extended from element-by-element exposure assessment towards comparative multielement profiling of anthropogenic underground environments. Combining controlled biological exposure, analytical quality screening, multivariate analysis, and robustness testing provides a framework for identifying consistent multielement signatures without requiring them to represent unique source fingerprints.

The conclusions remain restricted to the empirical MRU–TG comparison. A single site represented each underground-system category, passive environmental matrices were unavailable, and no parallel aerosol, dust, material, or gaseous-Hg measurements were performed. Replication across additional underground systems and integration with direct environmental measurements will be required to evaluate broader transferability and to link moss-bag signatures to specific transport processes and sources.

## Figures and Tables

**Table 1:** Final elemental concentrations (Cexp) and net concentration changes (NE) in moss bags exposed at underground MRU and TG points.

Element	MRU-P	MRU-D	TG-P	TG-D
**A. Final concentrations after exposure, Cexp**				
Mn	565 ± 280	449 ± 187	134 ± 17.1	115 ± 16.5
Fe	212 ± 44.4	241 ± 42.3	207 ± 71.4	231 ± 88.4
Cu	4.58 ± 0.711	4.87 ± 0.609	5.97 ± 0.802	6.14 ± 1.10
Zn	29.2 ± 4.96	31.4 ± 4.85	47.8 ± 14.0	39.3 ± 10.7
Pb	6.67 ± 2.66	6.61 ± 2.76	7.53 ± 1.35	6.92 ± 1.37
Hg	0.0361 ± 0.00500	0.0408 ± 0.00421	0.131 ± 0.0477	0.176 ± 0.0606
**B. Net changes relative to corresponding initial samples, NE**				
Mn	465 ± 280	374 ± 187	33.5 ± 17.1	39.0 ± 16.5
Fe	112 ± 44.4	135 ± 42.3	104 ± 71.4	113 ± 88.4
Cu	1.11 ± 0.711	1.12 ± 0.609	2.65 ± 0.802	2.28 ± 1.10
Zn	4.11 ± 4.96	11.7 ± 4.85	20.7 ± 14.0	19.4 ± 10.7
Pb	2.87 ± 2.66	2.60 ± 2.76	3.33 ± 1.35	2.64 ± 1.37
Hg	0.00280 ± 0.00500	0.00164 ± 0.00421	0.0999 ± 0.0477	0.139 ± 0.0606

Values are mean ± standard deviation (mg kg^−1^ dry matter). P, *Pleurozium schreberi*; D, *Dicranum polysetum*; Cexp, final concentration; NE, net change relative to corresponding initial material.

**Table 2:** Element-specific between-system effects and Site × Species interactions for net concentration changes at underground MRU and TG points.

Element	TG − MRU Estimate [95% CI]	q	Site × Species Interaction [95% CI]	q
Mn	−383 [−500, −267]	<0.001	97.2 [32.5, 162]	0.0136
Fe	−15.2 [−58.7, 28.3]	0.576	−14.1 [−39.0, 10.7]	0.255
Cu	1.35 [0.802, 1.90]	<0.001	−0.384 [−0.787, 0.0195]	0.0921
Zn	12.2 [5.89, 18.4]	<0.001	−8.78 [−12.7, −4.87]	<0.001
Pb	0.250 [−1.37, 1.87]	0.754	−0.431 [−1.07, 0.202]	0.210
Hg	0.117 [0.0960, 0.138]	<0.001	0.0401 [−0.00197, 0.0821]	0.0921

The site contrast is TG − MRU; negative estimates indicate higher NE at MRU. The interaction is [(D − P)TG − (D − P)MRU]. q-values are Benjamini–Hochberg adjusted separately for site and interaction effects.

## Data Availability

All data generated and analysed during this study are available from the corresponding author on reasonable request.

## Supplementary Materials

The following supporting information can be downloaded at: https://www.mdpi.com/article/10.3390/molecules31203727/s1, Supplementary: Method S1: Exposure design and sample handling; Method S2: Elemental determination and analytical quality control; Method S3: Data handling and statistical procedures. Figure S1: Study design and analytical workflow for comparative active moss-bag profiling in MRU and TG; Figure S2: Schematic layouts and exposure networks in the Boryszyn Loop, Międzyrzecz Fortified Region (MRU), and the Historic Silver Mine in Tarnowskie Góry (TG); Figure S3: Projection of outdoor samples onto the PCA space defined by underground NE profiles; Table S1: Analytical parameters for elemental determination; Table S2: Measured and certified concentrations in BCR-482 lichen (n = 5); Table S3: Initial analytical values and censoring information used in data handling; Table S4: Descriptive statistics for moss bags exposed at underground and outdoor MRU and TG points; Table S5: Element loadings and contributions in PCA; Table S6: Multivariate inference and cross-biomonitor concordance; Table S7: Element-specific mixed models and planned contrasts; Table S8: Robustness of element-specific between-system effects; Table S9: Dominant-element sensitivity of the underground MRU–TG multielement contrast.
