# Supplementary material for: Comparative Moss-Bag Biomonitoring of Multielement Signatures in Anthropogenic Underground Systems
Source: Molecules. 2026 Oct 17;31(20):3727. doi: 10.3390/molecules31203727 (PMC13707682; doi:10.3390/molecules31203727)
Supplement: Supplementary file 1 [file molecules-31-03727-s001.zip › molecules-4634573-supplementary.pdf]

Supplementary Material

Comparative Moss-Bag Biomonitoring of Multielement Signatures in Anthropogenic Underground Systems

Paweł Świsłowski [1]*, Grzegorz Kłys [1], Aleksandra Wilk [2], Joanna Chwiej [2], Małgorzata Rajfur [1]

Supplementary Methods

S1. Exposure design and sample handling

We established 20 exposure points at each site, with Pleurozium schreberi and Dicranum polysetum moss bags co-deployed at each point. MRU comprised 18 underground and two outdoor points, whereas TG comprised 14 underground and six outdoor points. Exposure lasted 90 days, from February to May 2025 at MRU and from March to June 2025 at TG. Bags were suspended approximately 1.5–2.0 m above the floor and at least 10 cm from walls or other surfaces. Outdoor samples were retained only as contextual observations because their number and environmental setting differed between sites; the primary inferential population comprised 64 underground moss-bag samples.

The moss material was mechanically cleaned before exposure, conditioned for 60 min in demineralised water (conductivity ≤1.0 µS cm^{-1}), and dried at 22–24 °C for 20–24 h. Flat nylon bags (10 × 10 cm; 2 mm mesh) contained 2.00 g dry moss, corresponding to 10.0 mg cm^{-2} external bag area. Initial (C_0) material underwent the same preparation and elemental determination pathway as the corresponding exposed material.

S2. Elemental determination and analytical quality control

We selected only green gametophyte material for analysis. For Mn, Fe, Ni, Cu, Zn, Cd and Pb, an 800 mg subsample was treated with 10 mL of 65% HNO_3 and 6 mL of 30% H_2O_2 and left for 24 h before microwave digestion using a Speedwave XPERT system. Digests were quantitatively transferred to 20 mL volumetric flasks and diluted with deionised water. No additional sample dilution was applied before F-AAS. We measured Mn, Fe, Ni, Cu, Zn, Cd, and Pb in the mineralised solutions using an iCE 3500 spectrometer (Thermo Fisher Scientific, USA).

Hg followed a separate analytical pathway and was determined directly in dry, non-mineralised moss material using an AMA 254 mercury analyser (Altec, Czech Republic). Each exposed sample was analysed in three instrumental replicates. F-AAS calibration used analytical standard solutions supplied by ANALYTIKA Ltd. We assessed analytical performance using BCR-482 lichen (n = 5).

S3. Data handling and statistical procedures

For each Site × Species combination, one initial moss sample was available, from which five subsamples were analysed. C_0 was calculated as the mean of these five subsample determinations. Within-sample variability of C_0 was expressed as the sample standard deviation calculated across the five subsample determinations (n = 5). Net concentration change was calculated as NE = Cexp − C_0. Negative NE values were retained and were not interpreted as negative deposition. Ni and Cd were excluded from continuous mixed-model and multivariate inference because of censoring at the operational LOQ; LOQ-coded observations were not treated as precise continuous concentrations.

Element-specific NE models used Site × Species fixed effects and a random intercept for physical exposure point. We compared equal and site-specific residual-variance

structures by AIC, and retained the site-specific structure when AIC decreased by at least two units. Benjamini–Hochberg correction was applied separately to the six Site, six Species and six Site × Species tests. Significant interactions were followed by planned site-within-species and species-within-site contrasts with Holm adjustment.

PCA used centred and standardised NE values. We performed site-level PERMANOVA/PERMDISP analyses separately for each species using Euclidean distances among standardised six-element profiles and 9,999 permutations. Species effects were evaluated separately within MRU and TG by paired PERMANOVA with label swaps restricted to the two species observations from the same Point_ID. Procrustes analysis with PROTEST assessed cross-species structural concordance. Robustness analyses included point-cluster bootstrap, leave-one-point-out analysis, sequential element exclusion (including simultaneous Mn + Hg exclusion), and point-cluster-robust models of log(Cexp).

Supplementary Tables

Table S1. Analytical parameters for elemental determination.

A. F-AAS parameters for Mn, Fe, Ni, Cu, Zn, Cd and Pb.

Element	Wavelength [nm]	IDL [mg L^{-1}]	IQL [mg L^{-1}]	Sample-equivalent IDL [mg kg^{-1}]	Sample-equivalent IQL [mg kg^{-1}]
Mn	279.5	0.0016	0.020	0.04	0.5
Fe	248.3	0.0043	0.050	0.1075	1.25
Ni	232.0	0.0043	0.050	0.1075	1.25
Cu	324.8	0.0045	0.033	0.1125	0.825
Zn	213.9	0.0033	0.010	0.0825	0.25
Cd	228.8	0.0028	0.013	0.07	0.325
Pb	217.0	0.0130	0.070	0.325	1.75

Sample-equivalent values were calculated from 0.800 g dry material diluted to 20 mL (factor = 25 L kg^{-1}). No additional sample dilution was applied.

B. Direct Hg determination by AMA 254.

Analyte	Measurement pathway	Wavelength [nm]	IDL	IQL
Hg	Direct determination in dry, non-mineralised moss	253.7	0.003 ng	0.010 ng

Hg was not determined in the digest; solution-based F-AAS conversion was therefore not applied.

Table S2. Measured and certified concentrations in BCR-482 lichen (n = 5).

Metal	BCR-482 lichen Concentration [mg/kg d.m.]	BCR-482 lichen ±Uncertainty [mg/kg d.m.]	Measured concentration Mean [mg/kg d.m.]	Measured concentration ±SD* [mg/kg d.m.]	Rel.** [%]
Mn	33.0	0.5	31.7	0.68	-3.9
Fe	804	160	771	154	-4.1
Ni	2.47	0.07	2.16	0.32	-13
Cu	7.03	0.19	6.63	0.17	-5.7
Zn	100.6	2.2	95.1	2.3	-5.5
Cd	0.56	0.02	0.53	0.03	-5.3
Hg	0.48	0.002	0.46	0.04	-4.4

Metal	BCR-482 lichen<br>Concentration<br>[mg/kg d.m.]	BCR-482 lichen<br>±Uncertainty<br>[mg/kg d.m.]	Measured concentration<br>Mean<br>[mg/kg d.m.]	Measured concentration<br>±SD*<br>[mg/kg d.m.]	Rel.**<br>[%]
Pb	40.9	1.4	38.2	1.0	-6.6

SD, standard deviation. Relative difference = 100 × (measured − certified)/certified.

Table S3. Initial analytical values and censoring information used in data handling.

A. Site- and species-matched initial concentrations (C_0; mean ± SD, n = 5 subsamples) used to calculate NE (mg kg^{-1} dry matter).

Element	MRU–P	MRU–D	TG–P	TG–D
Mn	100 ± 3.91	75.3 ± 1.51	101 ± 2.19	76.1 ± 2.11
Fe	100 ± 1.12	106 ± 5.59	103 ± 2.11	119 ± 4.55
Ni	1.25	1.25	1.25	1.25
Cu	3.47 ± 0.150	3.74 ± 0.196	3.32 ± 0.510	3.85 ± 0.219
Zn	25.1 ± 0.560	19.7 ± 0.559	27.1 ± 0.655	19.9 ± 0.695
Cd	0.638 ± 0.015	0.413 ± 0.049	0.620 ± 0.060	0.423 ± 0.054
Pb	3.81 ± 0.084	4.01 ± 0.041	4.19 ± 0.090	4.28 ± 0.040
Hg	0.0333 ± 0.002	0.0392 ± 0.003	0.0310 ± 0.001	0.0373 ± 0.002

B. Results recorded at or below the operational LOQ for Ni and Cd in the primary underground dataset.

Element	Site	At or below LOQ (n)	Total (n)	At or below LOQ (%)
Ni	MRU	29	36	80.6
Ni	TG	23	28	82.1
Cd	MRU	31	36	86.1
Cd	TG	2	28	7.14

Ni censoring did not differ between sites (Fisher exact p = 1.00), whereas Cd censoring was strongly asymmetric (Fisher exact p < 0.001). Ni and Cd were excluded from continuous mixed-model and multivariate inference.

Table S4. Descriptive statistics for moss bags exposed at underground and outdoor MRU and TG points (mg kg^{-1} dry matter).

A. Final concentrations after exposure at underground points, Cexp.

Element	Group	Mean	SD	Median	Q1	Q3	Min	Max
Mn	MRU–P	565	280	679	219	821	211	871
Mn	MRU–D	449	187	503	216	606	206	680
Mn	TG–P	134	17.1	136	125	142	101	169
Mn	TG–D	115	16.5	109	106	121	96.9	160
Fe	MRU–P	212	44.4	209	173	250	142	290
Fe	MRU–D	241	42.3	251	205	275	163	305
Fe	TG–P	207	71.4	189	170	208	161	439
Fe	TG–D	231	88.4	214	203	224	126	520
Cu	MRU–P	4.58	0.711	4.79	4.62	4.93	2.85	5.47
Cu	MRU–D	4.87	0.609	4.98	4.49	5.41	3.79	5.60
Cu	TG–P	5.97	0.802	5.96	5.39	6.40	4.57	7.34
Cu	TG–D	6.14	1.10	5.67	5.31	7.00	4.91	8.54

Element	Group	Mean	SD	Median	Q1	Q3	Min	Max
Zn	MRU–P	29.2	4.96	30.0	27.7	32.4	18.4	35.6
Zn	MRU–D	31.4	4.85	31.8	29.3	32.5	23.4	42.4
Zn	TG–P	47.8	14.0	42.7	41.8	47.1	33.5	91.2
Zn	TG–D	39.3	10.7	35.7	34.1	39.3	28.0	70.7
Pb	MRU–P	6.67	2.66	5.77	4.41	8.48	3.64	11.5
Pb	MRU–D	6.61	2.76	5.69	4.16	9.22	3.77	11.6
Pb	TG–P	7.53	1.35	7.50	6.84	7.74	5.54	11.0
Pb	TG–D	6.92	1.37	6.71	5.93	7.47	5.06	9.86
Hg	MRU–P	0.0361	0.00500	0.0359	0.0338	0.0387	0.0288	0.0499
Hg	MRU–D	0.0408	0.00421	0.0400	0.0385	0.0412	0.0357	0.0538
Hg	TG–P	0.131	0.0477	0.121	0.0927	0.152	0.0673	0.212
Hg	TG–D	0.176	0.0606	0.185	0.130	0.227	0.0516	0.242

B. Net changes relative to corresponding initial samples at underground points, NE.

Element	Group	Mean	SD	Median	Q1	Q3	Min	Max
Mn	MRU–P	465	280	579	119	721	111	771
Mn	MRU–D	374	187	428	140	531	131	604
Mn	TG–P	33.5	17.1	34.7	23.6	41.4	−0.233	68.5
Mn	TG–D	39.0	16.5	33.0	30.3	45.1	20.8	84.2
Fe	MRU–P	112	44.4	108	72.7	150	42.0	190
Fe	MRU–D	135	42.3	145	98.8	169	56.6	199
Fe	TG–P	104	71.4	85.7	67.2	104	57.8	336
Fe	TG–D	113	88.4	95.3	84.4	106	7.04	402
Cu	MRU–P	1.11	0.711	1.32	1.16	1.47	−0.610	2.01
Cu	MRU–D	1.12	0.609	1.23	0.749	1.66	0.0500	1.86
Cu	TG–P	2.65	0.802	2.65	2.08	3.08	1.25	4.03
Cu	TG–D	2.28	1.10	1.82	1.46	3.15	1.06	4.69
Zn	MRU–P	4.11	4.96	4.92	2.64	7.38	−6.69	10.5
Zn	MRU–D	11.7	4.85	12.1	9.60	12.8	3.67	22.6
Zn	TG–P	20.7	14.0	15.5	14.7	20.0	6.43	64.1
Zn	TG–D	19.4	10.7	15.9	14.3	19.4	8.19	50.8
Pb	MRU–P	2.87	2.66	1.96	0.604	4.67	−0.170	7.70
Pb	MRU–D	2.60	2.76	1.69	0.152	5.22	−0.240	7.62
Pb	TG–P	3.33	1.35	3.31	2.64	3.55	1.35	6.78
Pb	TG–D	2.64	1.37	2.43	1.65	3.19	0.778	5.58
Hg	MRU–P	0.00280	0.00500	0.00253	0.000448	0.00537	−0.00455	0.0165
Hg	MRU–D	0.00164	0.00421	0.000845	−0.000643	0.00202	−0.00345	0.0146
Hg	TG–P	0.0999	0.0477	0.0902	0.0617	0.121	0.0363	0.181
Hg	TG–D	0.139	0.0606	0.148	0.0932	0.190	0.0143	0.204

C. Net changes relative to corresponding initial samples at outdoor points, NE.

Element	MRU–P	MRU–D	TG–P	TG–D
Mn	115 [115–116]	133 [132–134]	29.3 [−10.8–48.9]	50.4 [18.1–69.8]
Fe	128 [125–130]	128 [125–131]	205 [36.9–285]	263 [166–342]
Cu	1.52 [1.47–1.58]	1.54 [1.32–1.76]	4.74 [0.530–7.85]	5.64 [1.51–11.2]
Zn	3.78 [3.18–4.38]	11.7 [11.5–11.9]	68.2 [5.50–93.1]	81.0 [32.9–116]
Pb	8.22 [8.19–8.24]	7.43 [7.41–7.44]	22.8 [7.09–37.7]	26.8 [11.5–47.7]
Hg	0.00264 [0.00162–0.00367]	−0.000442 [−0.00103–0.000148]	0.0259 [0.00990–0.0643]	0.0126 [0.00519–0.0213]

P, Pleurozium schreberi; D, Dicranum polysetum; Q1 and Q3, first and third quartiles. Outdoor values are presented as mean [min–max]. Negative NE values were retained and were not interpreted as negative deposition.

Table S5. Element loadings and contributions in PCA.

Element	Loading PC1	Share in PC1 (%)	Loading PC2	Share in PC2 (%)
Mn	0.518	26.8	0.323	10.4
Fe	0.0718	0.515	0.763	58.3
Cu	−0.471	22.2	0.111	1.23
Zn	−0.409	16.8	0.480	23.0
Pb	−0.275	7.57	−0.252	6.36
Hg	−0.512	26.2	0.0833	0.693

PC1 explained 44.4% and PC2 22.6% of total variance. Axis signs are arbitrary; PC1 was oriented with positive Mn and PC2 with positive Fe.

Table S6. Multivariate inference and cross-biomonitor concordance.

A. PERMANOVA and PERMDISP results for the MRU–TG site contrast.

Species	pseudo-(F)	R2	PERMANOVA p	PERMDISP F	PERMDISP p
Pleurozium schreberi	17.4	0.367	<0.001	0.0912	0.778
Dicranum poly-setum	14.0	0.318	<0.001	0.564	0.488

B. Within-system species effects and cross-biomonitor structural concordance.

System	Paired PER-MANOVA pseudo-F	Species R^2	PERMANOVA p	Procrustes r	PROTEST p
MRU	3.16	0.0849	<0.001	0.783	<0.001
TG	1.17	0.0432	0.0265	0.771	<0.001

Species R^2 values derive from separate paired within-system PERMANOVA analyses and are not direct partitions from the site-level PERMANOVA models. Procrustes coefficients describe agreement in relative point configuration, not equality of absolute NE values.

Table S7. Element-specific mixed models and planned contrasts.

A. Full fixed-effect results from element-specific linear mixed-effects models.

Element	Effect	Estimate	95% CI	p	q	Residual structure
Mn	Site: TG – MRU	−383	−500 to −267	1.85×10^{-7}	5.56×10^{-7}	Site-specific
Mn	Species: D – P	−43.0	−75.4 to −10.7	0.0108	0.0217	Site-specific
Mn	Interaction	97.2	32.5 to 162	0.00454	0.0136	Site-specific
Fe	Site: TG – MRU	−15.2	−58.7 to 28.3	0.480	0.576	Equal
Fe	Species: D – P	15.4	2.98 to 27.8	0.0168	0.0252	Equal
Fe	Interaction	−14.1	−39.0 to 10.7	0.255	0.255	Equal
Cu	Site: TG – MRU	1.35	0.802 to 1.90	2.21×10^{-5}	4.42×10^{-5}	Equal

Element	Effect	Estimate	95% CI	p	q	Residual structure
Cu	Species: D − P	−0.178	−0.380 to 0.0236	0.0815	0.0815	Equal
Cu	Interaction	−0.384	−0.787 to 0.0195	0.0614	0.0921	Equal
Zn	Site: TG − MRU	12.2	5.89 to 18.4	0.000419	0.000629	Equal
Zn	Species: D − P	3.17	1.22 to 5.13	0.00242	0.0125	Equal
Zn	Interaction	−8.78	−12.7 to −4.87	7.46×10^{-5}	0.000448	Equal
Pb	Site: TG − MRU	0.250	−1.37 to 1.87	0.754	0.754	Site-specific
Pb	Species: D − P	−0.481	−0.798 to −0.164	0.00417	0.0125	Site-specific
Pb	Interaction	−0.431	−1.07 to 0.202	0.175	0.210	Site-specific
Hg	Site: TG − MRU	0.117	0.0960 to 0.138	2.47×10^{-12}	1.48×10^{-11}	Site-specific
Hg	Species: D − P	0.0189	−0.00215 to 0.0399	0.0767	0.0815	Site-specific
Hg	Interaction	0.0401	−0.00197 to 0.0821	0.0610	0.0921	Site-specific

All tests had df = 30. The interaction is [(D − P)TG − (D − P)MRU]. Benjamini–Hochberg q-values were calculated separately for Site, Species and interaction effects. The final column reports the selected residual structure.

B. Planned simple contrasts for Mn and Zn following significant Site × Species interactions.

Element	Contrast type	Contrast	Estimate [95% CI] (mg kg^{-1} d.m.)	Holm-adjusted p
Mn	Site within species	TG − MRU, P	−432 [−553, −311]	<0.001
Mn	Site within species	TG − MRU, D	−335 [−455, −214]	<0.001
Mn	Species within site	D − P, MRU	−91.6 [−155, −28.3]	0.0121
Mn	Species within site	D − P, TG	5.57 [−7.59, 18.7]	0.395
Zn	Site within species	TG − MRU, P	16.5 [9.99, 23.1]	<0.001
Zn	Site within species	TG − MRU, D	7.76 [1.20, 14.3]	0.0219
Zn	Species within site	D − P, MRU	7.56 [4.98, 10.1]	<0.001
Zn	Species within site	D − P, TG	−1.22 [−4.15, 1.71]	0.402

P, P. schreberi; D, D. polysetum. p-values were adjusted within each pair of planned contrasts using the Holm method.

Table S8. Robustness of element-specific between-system effects.

A. Cluster-bootstrap and leave-one-point-out assessment.

Element	TG–MRU estimate [bootstrap 95% CI]	q	LOO range	Interaction estimate [bootstrap 95% CI]	q	LOO range
Mn	−383 [−485, −279]	<0.001	−401 to −369	97.2 [41.9, 154]	0.0136	84.5 to 108
Fe	−15.2 [−51.8, 34.8]	0.576	−35.3 to −9.70	−14.1 [−36.8, 9.16]	0.255	−19.3 to −8.95

Element	TG–MRU estimate [bootstrap 95% CI]	q	LOO range	Interaction estimate [bootstrap 95% CI]	q	LOO range
Cu	1.35 [0.836, 1.90]	<0.001	1.22 to 1.45	−0.384 [−0.763, 0.0118]	0.0921	−0.487 to −0.315
Zn	12.2 [6.82, 19.5]	<0.001	9.27 to 12.8	−8.78 [−12.5, −4.97]	<0.001	−9.68 to −7.86
Pb	0.250 [−1.14, 1.56]	0.754	0.00429 to 0.540	−0.431 [−1.03, 0.164]	0.210	−0.596 to −0.317
Hg	0.117 [0.0965, 0.138]	<0.001	0.111 to 0.122	0.0401 [0.000324, 0.0734]	0.0921	0.0342 to 0.0546

Bootstrap percentile confidence intervals used 20,000 point-level resamples within each site, retaining paired species observations. LOO ranges are minimum and maximum estimates after sequential removal of one exposure point.

B. Point-cluster-robust sensitivity analysis of log-transformed final concentrations (Cexp).

Element	TG/MRU ratio [95% CI]	q	Site × Species ratio-of-ratios [95% CI]	q
Mn	0.279 [0.215–0.362]	<0.001	1.023 [0.898–1.165]	0.732
Fe	0.945 [0.803–1.112]	0.495	0.968 [0.860–1.090]	0.711
Cu	1.282 [1.156–1.421]	<0.001	0.955 [0.884–1.031]	0.472
Zn	1.410 [1.229–1.617]	<0.001	0.762 [0.688–0.845]	<0.001
Pb	1.152 [0.942–1.408]	0.203	0.933 [0.858–1.013]	0.300
Hg	3.716 [3.167–4.359]	<0.001	1.167 [0.862–1.579]	0.476

Ratios are exponentiated equal-weight marginal contrasts from log(Cexp) models. Values >1 indicate higher final concentrations in TG; values <1 indicate higher final concentrations in MRU.

Table S9. Dominant-element sensitivity of the underground MRU–TG multielement contrast.

Variant	Species	Elements retained	pseudo-F	R^2	PERMANOVA p	PERMDISP F	PERMDISP p
Full profile	P	Mn, Fe, Cu, Zn, Pb, Hg	17.36	0.367	<0.001	0.0912	0.778
Full profile	D	Mn, Fe, Cu, Zn, Pb, Hg	13.96	0.318	<0.001	0.564	0.488
Without Mn	P	Fe, Cu, Zn, Pb, Hg	15.11	0.335	<0.001	0.354	0.578
Without Mn	D	Fe, Cu, Zn, Pb, Hg	10.64	0.262	<0.001	2.13	0.164
Without Fe	P	Mn, Cu, Zn, Pb, Hg	23.46	0.439	<0.001	0.593	0.455
Without Fe	D	Mn, Cu, Zn, Pb, Hg	18.02	0.375	<0.001	0.134	0.725
Without Cu	P	Mn, Fe, Zn, Pb, Hg	15.10	0.335	<0.001	0.354	0.577
Without Cu	D	Mn, Fe, Zn, Pb, Hg	13.85	0.316	<0.001	0.00003	0.995

Variant	Species	Elements retained	pseudo-F	R^2	PER-MANOVA p	PERMDISP F	PERMDISP p
Without Zn	P	Mn, Fe, Cu, Pb, Hg	16.56	0.356	<0.001	1.47	0.255
Without Zn	D	Mn, Fe, Cu, Pb, Hg	15.53	0.341	<0.001	0.00162	0.975
Without Pb	P	Mn, Fe, Cu, Zn, Hg	23.34	0.438	<0.001	1.13	0.334
Without Pb	D	Mn, Fe, Cu, Zn, Hg	18.47	0.381	<0.001	3.96	0.0451
Without Hg	P	Mn, Fe, Cu, Zn, Pb	12.70	0.297	<0.001	1.22	0.284
Without Hg	D	Mn, Fe, Cu, Zn, Pb	8.96	0.230	<0.001	0.0471	0.842
Without Mn + Hg	P	Fe, Cu, Zn, Pb	9.51	0.241	<0.001	0.0582	0.820
Without Mn + Hg	D	Fe, Cu, Zn, Pb	4.83	0.139	<0.001	0.836	0.399

Each analysis used standardised NE values separately for each species. The D. polysetum analysis after Pb exclusion showed borderline dispersion heterogeneity (PERMDISP p = 0.0451); the central simultaneous Mn + Hg exclusion retained non-significant PERMDISP for both species.

Supplementary Figures

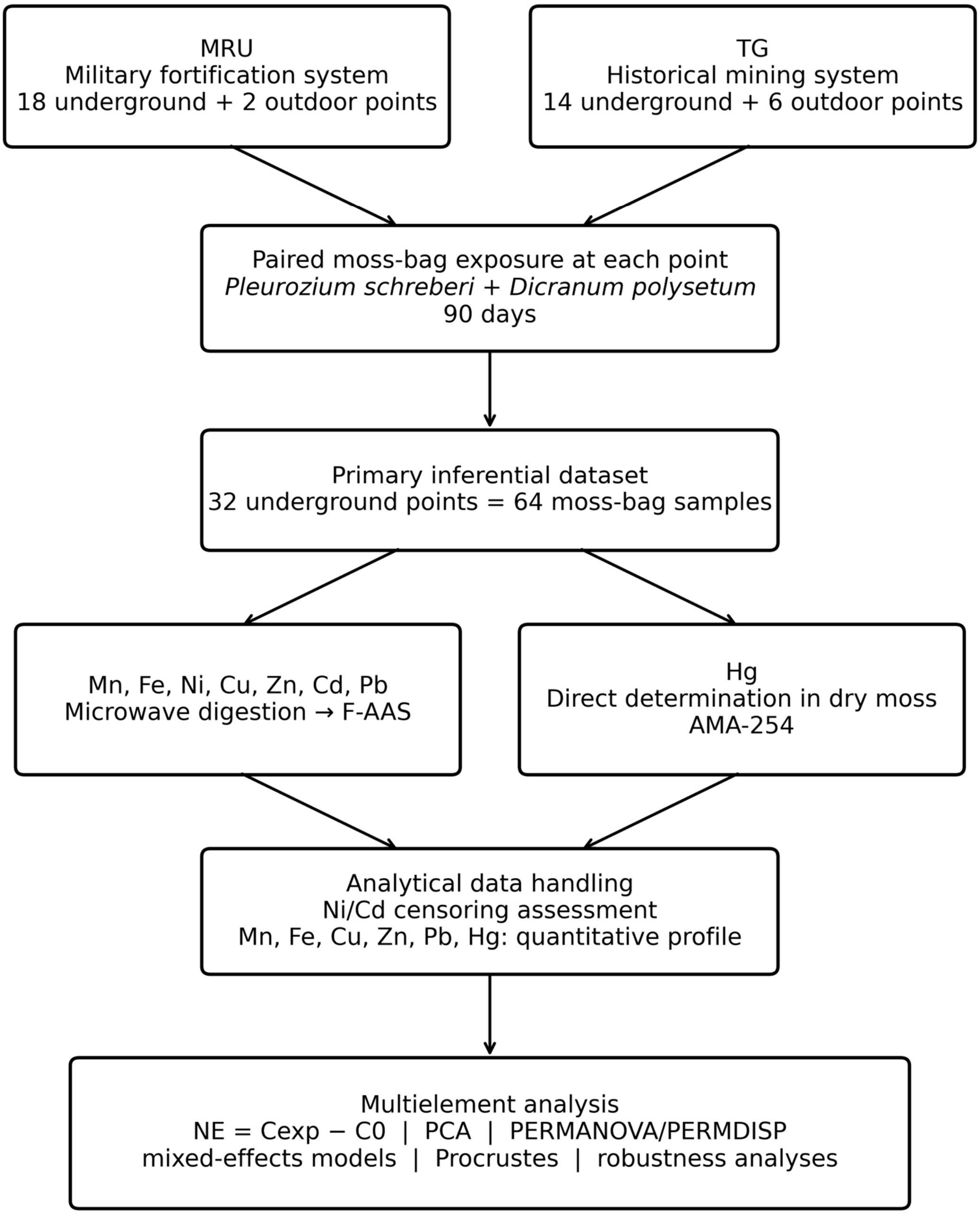

Figure S1. Study design and analytical workflow for comparative active moss-bag profiling in MRU and TG.

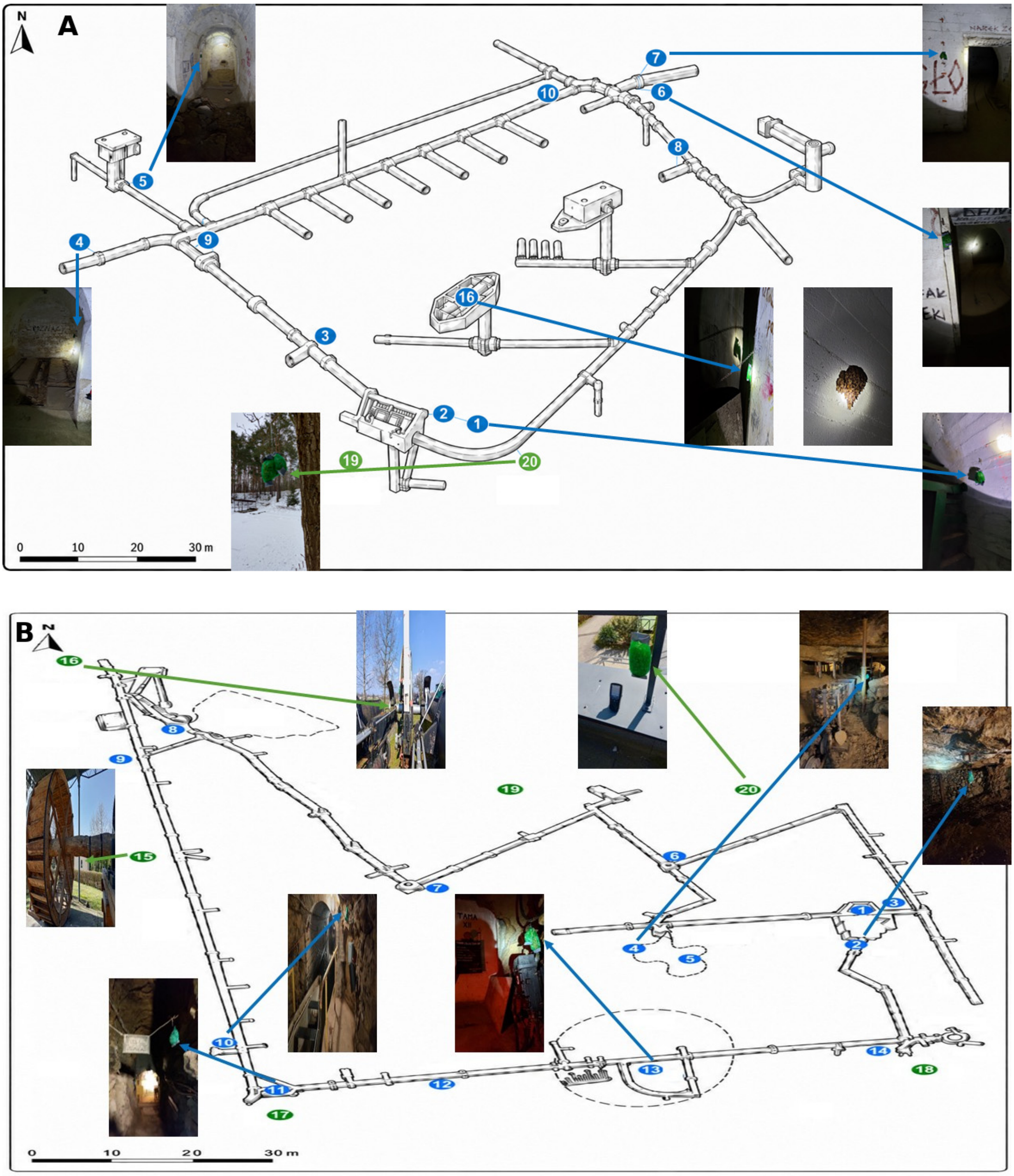

Figure S2. Schematic layouts and exposure networks in (A) the Boryszyn Loop, Międzyrzecz Fortified Region (MRU), and (B) the Historic Silver Mine in Tarnowskie Góry (TG). Blue numbers denote underground exposure points and green numbers denote outdoor points; photographs provide site context.

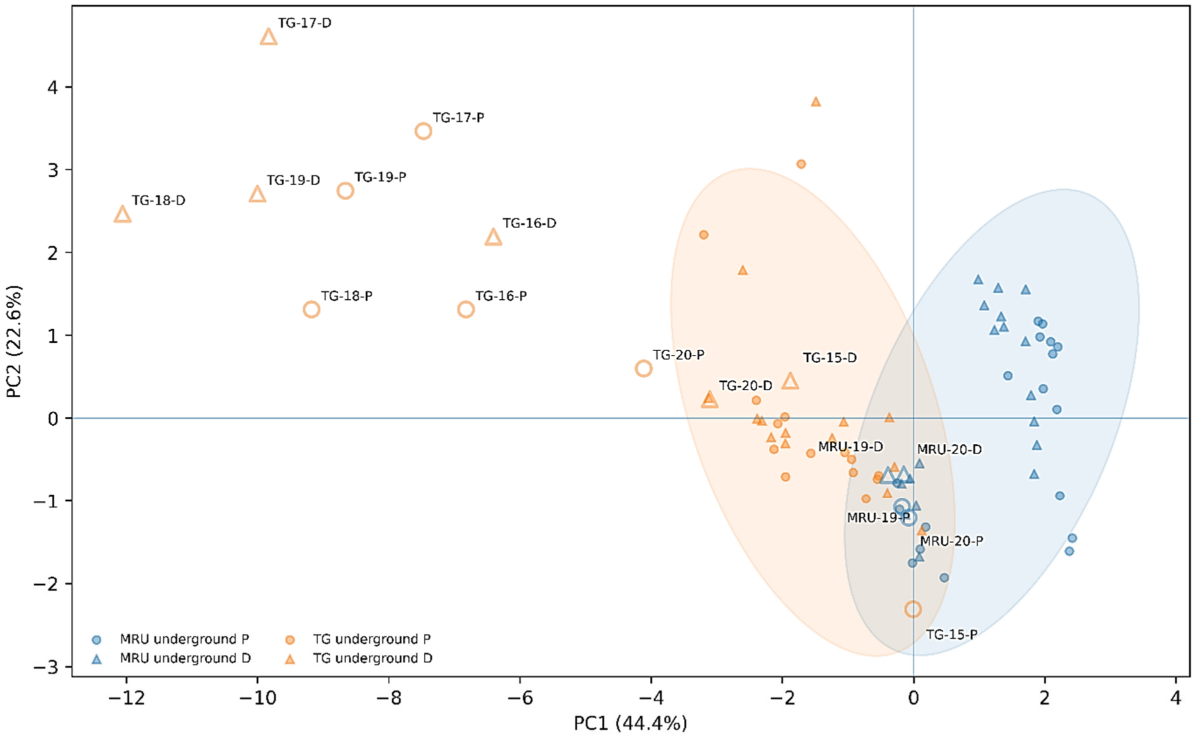

Figure S3. Projection of outdoor samples onto the PCA space defined by underground NE profiles. Filled symbols are underground samples; open symbols are outdoor samples projected with the centring, scaling and loadings fitted to underground data. Colours denote site and shapes denote moss species. Ellipses describe underground samples only.
